# Dorsal root ganglion neurons recapitulate the traumatic axonal injury of CNS neurons in response to a rapid stretch *in vitro*

**DOI:** 10.3389/fncel.2023.1111403

**Published:** 2023-03-29

**Authors:** Alexandra A. Adams, Ying Li, Haesun A. Kim, Bryan J. Pfister

**Affiliations:** ^1^Center for Injury Biomechanics, Materials and Medicine, Department of Biomedical Engineering, New Jersey Institute of Technology, Newark, NJ, United States; ^2^Department of Biological Sciences, Rutgers University Newark, Newark, NJ, United States

**Keywords:** traumatic axonal injury (TAI), stretch injury, sodium channel proteolysis, calcium homeostasis, axonal degeneration

## Abstract

**Introduction:** *In vitro* models of traumatic brain injury (TBI) commonly use neurons isolated from the central nervous system. Limitations with primary cortical cultures, however, can pose challenges to replicating some aspects of neuronal injury associated with closed head TBI. The known mechanisms of axonal degeneration from mechanical injury in TBI are in many ways similar to degenerative disease, ischemia, and spinal cord injury. It is therefore possible that the mechanisms that result in axonal degeneration in isolated cortical axons after *in vitro* stretch injury are shared with injured axons from different neuronal types. Dorsal root ganglia neurons (DRGN) are another neuronal source that may overcome some current limitations including remaining healthy in culture for long periods of time, ability to be isolated from adult sources, and myelinated *in vitro*.

**Methods:** The current study sought to characterize the differential responses between cortical and DRGN axons to mechanical stretch injury associated with TBI. Using an *in vitro* model of traumatic axonal stretch injury, cortical and DRGN neurons were injured at a moderate (40% strain) and severe stretch (60% strain) and acute alterations in axonal morphology and calcium homeostasis were measured.

**Results:** DRGN and cortical axons immediately form undulations in response to severe injury, experience similar elongation and recovery within 20 min after the initial injury, and had a similar pattern of degeneration over the first 24 h after injury. Additionally, both types of axons experienced comparable degrees of calcium influx after both moderate and severe injury that was prevented through pre-treatment with tetrodotoxin in cortical neurons and lidocaine in DRGNs. Similar to cortical axons, stretch injury also causes calcium activated proteolysis of sodium channel in DRGN axons that is prevented by treatment with lidocaine or protease inhibitors.

**Discussion:** These findings suggest that DRGN axons share the early response of cortical neurons to a rapid stretch injury and the associated secondary injury mechanisms. The utility of a DRGN *in vitro* TBI model may allow future studies to explore TBI injury progression in myelinated and adult neurons.

## Introduction

The initiating event in traumatic brain injury (TBI) is a mechanical loading to the head (Holbourn, 1945; Smith and Meaney, 2000; Pfister et al., 2003). In closed head injuries, traumatic damage to neurons and their axons is thought to occur from rapid stretching of the brain tissue as a result of damaging head motions (Adams et al., 1984; Grady et al., 1993; Smith et al., 2003; Johnson et al., 2013). *In vitro* models that induce a controlled rapid stretch to neuronal cultures are frequently used to investigate the progression of secondary injury processes in real time (Pfister et al., 2003; Magou et al., 2011; Morrison et al., 2011). These models have been in use for many years and replicate morphological and ultrastructural changes observed *in vivo*, including alterations in neurofilament structure, axolemmal permeability, and axonal swelling formation (Smith et al., 1999; Pfister et al., 2003, 2004; Iwata et al., 2004; Cullen et al., 2011). They have had a significant impact in the field by identifying important biological processes involved in axonal pathology including an immediate rise in intracellular calcium levels, protease activation, and axonal degeneration (Galbraith et al., 1993; Ellis et al., 1995; Cargill and Thibault, 1996; LaPlaca and Thibault, 1997; Tavalin et al., 1997; Smith et al., 1999; Di et al., 2000; Wolf et al., 2001; Pfister et al., 2003, 2004; Goforth et al., 2004; Kao et al., 2004; Morrison et al., 2006). These pathologies have been associated with Diffuse Axonal Injury (DAI) in humans.

*In vitro* neuronal stretch injury models typically use mixed embryonic cortical or hippocampal primary cultures (Lusardi et al., 2004; Magou et al., 2015). Dorsal root ganglion neurons (DRGN) are another source of primary neurons that may allow for experimental questions that cannot currently be addressed with cortical cultures. For instance, DAI is a pathology in myelinated axons and there are currently no robust methods to myelinate cortical or hippocampal neurons in culture. Unlike cortical neuronal sources, DRGNs can be myelinated with Schwann cells or oligodendrocytes *in vitro*, they can survive for months in culture, and neurons from adult sources can be successfully cultured (Huang et al., 2009; Liu et al., 2013; Heffernan and Maurel, 2018; Loverde et al., 2020).

DRGNs are considered neurons of the peripheral nervous system (PNS), with an axon extending into the periphery and an axon that extends into the spinal cord of the central nervous system (CNS; Hoffman, 2010). While DRGN and cortical neurons are different in many ways, the progression of neuronal degeneration can be quite similar among neurological diseases and injuries including Alzheimer’s disease, ischemia, and spinal cord injury (SCI; Stys, 1998; Johnson et al., 2010; Ye et al., 2012). Here, we were interested in establishing whether the acute effects of traumatic axon stretch injury in DRGN are comparable to the injury response of cortical neurons. This study examines whether DRGN and cortical axons respond similarly to the primary mechanical stretch and develop similar secondary injury mechanisms within the first 24 h post-injury including morphological changes, calcium influx, and sodium channel proteolysis.

## Methods

### Stretch-injury device

**Figure 1:**
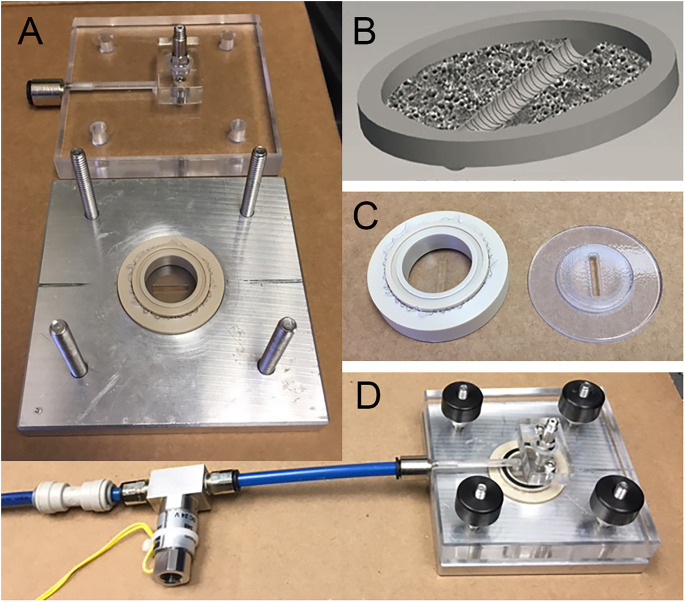
Axonal stretch injury. **(A)** Injury device pressure chamber and culture well. **(B)** Neuronal cultures are grown on a silicone elastic membrane. A region of isolated axons is formed by creating a 2 mm cell free strip across the culture. During the development of the cultures, DRGN and cortical axons transverse the cell free gap. **(C)** Uniaxial stretch of axons is achieved by placing a rigid mask under the silicone membrane to allow deformation to only the 2 mm strip of axons. **(D)** Assembled stretch injury system.

Neurons were cultured on silicone elastic membranes (Specialty Manufacturing Inc., Saginaw, MI) and deformed with the application of a pressure-pulse of air (Magou et al., 2011; Figure 1). This rapid deformation causes the stretching of neurons that are adherent to the substrate. In this study, we applied a uniaxial stretch to isolated axon tracts spanning a 2 mm cell free gap. In order to injure axons crossing the cell free gap, a rigid mask printed from acrylonitrile butadiene styrene (ABS) plastic with a 2-mm gap in the center was placed underneath the well prior to injury. Masks were printed using a three-dimensional rapid prototyping system (SST 1200es, Dimension, Inc., Eden Prairie, MN) designed using Pro-Engineer software (PTC, Needham, MA).

Uniaxial stretch injury to axons was measured in terms of strain, defined by the amount the membrane is stretched divided by its original length. The rate of injury was measured in terms of the strain rate, defined by the applied strain over the rise time to maximum strain (i.e., a 60% strain applied over a period of 20 ms has a strain rate of 30 s^−1^). Axons were injured at uniaxial strains of 40 or 60% at a common strain rate of 30 s^−1^. These injury parameters have been experimentally determined to induce the formation of undulations immediately post injury and axonal swellings within 2 h after injury in cortical axons. Both cortical and DRGN axons were injured between 10 and 12 days* in vitro* (DIV) at both strains.

### Neuronal cultures

Custom polyetheretherketone (PEEK) wells assembled with elastic silicone membranes were submerged in dH_2_O and autoclaved for 1 h. For cortical cultures, wells were coated with poly-L-lysine (0.05 mg/ml; Peptides International, Louisville, KY) and incubated at 37°C overnight for optimal adsorption into the surface of the silicone. On the day of isolation, wells were rinsed three times with sterile dH_2_O and left to dry prior to plating. For DRGN cultures, wells were coated with 10 μg/ml high molecular weight poly-D-lysine (BD Biosciences, Bedford, MA) in phosphate buffered saline (PBS) for 1 h. Wells were then rinsed three times and coated with Matrigel (1:35 dilution, BD Biosciences, Bedford, MA) extracellular matrix overnight, then aspirated and allowed to dry the following day prior to plating.

In order to create a cell free zone for uniaxial stretching of isolated axons, silicone sheeting was cut into strips of 2 mm in width to fit the size of the gap in injury masks. After drying, 2 mm wide silicone strips were placed at the center of each well using a sterilized deformation mask for placement guidance. On the day after plating cells (DIV 1), strips were removed to create a cell free zone. Developing axons traverse the gap creating isolated axons for stretch injury. Cortical axons completely integrate across the 2 mm gap within 10 DIV (Iwata et al., 2004; Monnerie et al., 2010). Here it was found that DRGN axons also completely integrate across the gap by 10 DIV, the time at which both cultures were injured.

For cortical cultures, cortices were isolated from E17 Sprague-Dawley rat pups (Kingston, NY), the meninges were removed and cortices were stored on ice in Hanks Balanced Buffer Solution (HBSS) containing Ca^2+^ and Mg^2+^ followed by a brief calcium switch in Ca^2+^ and Mg^2+^ free HBSS. Tissue was then digested in 0.025% Trypsin containing DNase I (1.0 mg/ml) and EDTA (0.2 g/L) for 30 min in the incubator at 37°C. Trypsin solution was then removed and neutralized using 10% fetal bovine serum (FBS) in Ca^2+^ and Mg^2+^ free HBSS. After removal of FBS, cells were suspended in 500 μl of cortical growth medium [Neurobasal media, 2% B-27, 1% penicillin-streptomycin, and 0.4 mM L-glutamine (Thermo Fisher Scientific, Waltham, MA)]. Cells in the growth medium were then centrifuged at 2,000 RPM for 5 min. The supernatant was removed, and cells were resuspended in 500 μl of media. After the addition of 5 ml growth media, cells were filtered through a sterilized nylon mesh of 100 μm pore size, followed by 50 μm. Cells were then counted and plated at a density of 375,000 cells/cm^2^.

For DRGN cultures, spinal cords were removed from E17 Sprague-Dawley rat pups and stored in ice cold Leibovitz’s L-15 medium. DRGNs were removed from the spinal cord, collected, and incubated in trypsin for 1 h at 37°C. Following tissue digestion, trypsin was removed and replaced with 100% FBS and centrifuged at 6,000 RPM for 10 min. The supernatant was removed, and cells were resuspended in a growth medium (Neurobasal medium, 2% B-27, 1% penicillin-streptomycin, 0.4 mM L-glutamine, 500 μl 20% D-glucose, 20 ng/ml nerve growth factor, 20 μM 5-fluoro-2’-deoxyuridine + 20 μM Uridine). Cells were plated within 100 μl droplets on both sides of the silicone strip and allowed to attach for 1 h. After 1 h, an additional 500 μl of growth media was added to each well. Media was changed after 24 h and every three days following. After 3 days in culture, cells were maintained in growth media without mitotic inhibitors. Media was changed 24 h after isolation and again every three days following. All animal work conforms to the National Research Council’s Guide for the Care and Use of Laboratory Animals and protocols were approved by the Rutgers-University Newark Animal Institutional Animal Care and Use Committee.

### Measurement of axonal deformation

Axonal morphological alterations in the region of injury were analyzed in two ways: immediate formation of undulations and the time frame for recovery to the original axon length. Phase contrast time-lapse imaging was used to record changes in axonal length over 20 min after injury with one picture taken every min of the recording. Undulatory deformations were characterized immediately after stretch and quantified by percent strain:


(L(t)−L0)/L0 ∗ 100

where L_0_ is the original length of the segment, and L(t) is the length of the segment at time ‘t’. Percent recovery was determined similarly:


[1− (L(t)−L0)/L0] ∗ 100

where 100% would represent the complete recovery of strain to the original axon length L_0_.

To assess degeneration, a region of interest was selected within the unaxially stretched area of isolated axons prior to the start of the experiments. Images were captured prior to injury (0 h) and at 1, 2, 4, and 24 h post injury. At each time point, images within the region of interest were visually compared to the corresponding photo taken before injury. Images were assessed based on the presence or absence of the following characteristics: the development of axonal swellings along the length of injured axons; and degenerating axons, characterized by visible disconnection of a portion of the axon or complete loss of the axon within the field of view (Smith and Meaney, 2000). Six DRGN and eight cortical cultures from three different tissue preparations injured at 60% strain as well as eleven DRGN and six cortical cultures from three additional preparations injured at 40% strain were included in morphological analyses.

### Calcium imaging

Changes in intracellular calcium were measured from injured cortical and DRGN axons. Cells were gently rinsed three times with HBSS containing Ca^2+^ and Mg^2+^ and loaded with 4 μM Fluo-4 AM (F-14201, Invitrogen) solubilized in 100% anhydrous dimethylsulfoxide (DMSO) with Pluronic F-127 cellular detergent (0.01% w/v). Stock dye was diluted in a controlled saline solution (CSS; 120 mM NaCl, 5.4 mM KCl, 0.8 mM MgCl_2_, 1.8 mM CaCl_2_, 15 mM glucose, 25 mM HEPES at pH 7.4; Smith et al., 1999). Cells were incubated in loading solution for 30 min. The loading solution was then removed and replaced with fresh CSS to promote de-esterification of the dye. Prior to injury, cultures were rinsed one time with CSS for 3 min.

Fluorescent intensity was captured using a Nikon Eclipse TE2000 inverted microscope with a 20× objective in conjunction with a 75 watt xenon arc lamp and Photometrics CoolSNAP EZ CCD Camera. All camera settings were adjusted at the beginning of the experiment during the baseline measurement and not adjusted further. To minimize photobleaching of cells and increase light sensitivity, 2 × 2 camera binning was used. Automatic image acquisition was programmed using NIS Elements software (Nikon Instruments, Inc, Melville, NY) and controlled the shutter (Lambda SC Smart Shutter, Sutter Instruments, Novato, CA). Images were collected at 2 s intervals for thirty seconds of baseline measurements prior to injury followed by 2 min of recording post-injury and then at 1-min intervals for the following 5 min.

Fluorescent intensity was measured in individual axons using ImageJ software. The “StackReg” plugin was used to correct displacement between frames during and after injury (Thévenaz et al., 1998). Individual axons were outlined using freehand selection in ImageJ and mean gray value measurements were taken from each selected region (each axon) in each frame of the recording. Three measurements of background intensity were taken in each frame of the recordings, averaged, and subtracted from the intensity measurements (Schneider et al., 2012). Changes in fluorescent intensity were reported in terms of the ratio of the measured fluorescence at each time point (F) divided by the mean baseline fluorescence (F_0_), where baseline intensity by definition has a value of F/F_0_ = 1.

The dose for lidocaine blockage of sodium channels in DRGNs was determined experimentally based on previous findings (Roy and Narahashi, 1992). Cultures were pre-treated with lidocaine after loading and rinsing of Fluo-4 calcium indicator dye and before stretch injury. We found that a 200 μM concentration of lidocaine was sufficient to block both TTX sensitive and resistant sodium channels under injury of 60% strain at a rate of 30 s^−1^. To verify that extracellular calcium enters the axon, cultures were injured using HBSS free of Ca^2+^ and Mg^2+^ to rinse calcium and magnesium from the extracellular environment.

### Immunocytochemistry

At 2 h post-injury, DRGN cultures were rinsed with phosphate-buffered solution (PBS) and fixed for 20 min in 4% paraformaldehyde. Axons were then permeabilized with 0.3% Triton-X, blocked with 3% bovine serum albumin, and immunostained with a rabbit anti-neurofilament (NF200; 1:500; Sigma-Aldrich, St. Louis, MO), rabbit polyclonal anti-NaCh brain type II (epitope corresponds to the I-II intracellular loop; 1:50; Sigma-Aldrich, St. Louis, MO), and rabbit polyclonal anti-NaCh pan (epitope corresponds to the III-IV loop; Nav1, 1:100; Sigma-Aldrich, St. Louis, MO). The next day, samples were rinsed three times in 5 min intervals with phosphate buffered saline (PBS) before incubation in Alexa 488 or 594 secondary antibodies for 1 h. Samples were then rinsed three times in 5 min intervals before cutting silicone from the wells and mounting on glass slides.

### Statistical analysis

Data expressed as the mean ± the standard error of the mean. SPSS (IBM, Armonk, NY) statistical software was used for analysis. Differences between the means of cortical and DRGN morphological features were analyzed by the student’s *T*-test. Calcium measurement data were analyzed with the Kruskal-Wallis nonparametric test due to the data not meeting the assumptions of normality or homogeneity of variances using Levene’s test. *Post hoc* pairwise comparison was carried out between groups with significance values adjusted by the Bonferroni correction for multiple tests.

## Results

### Axon undulation and recovery

**Figure 2:**
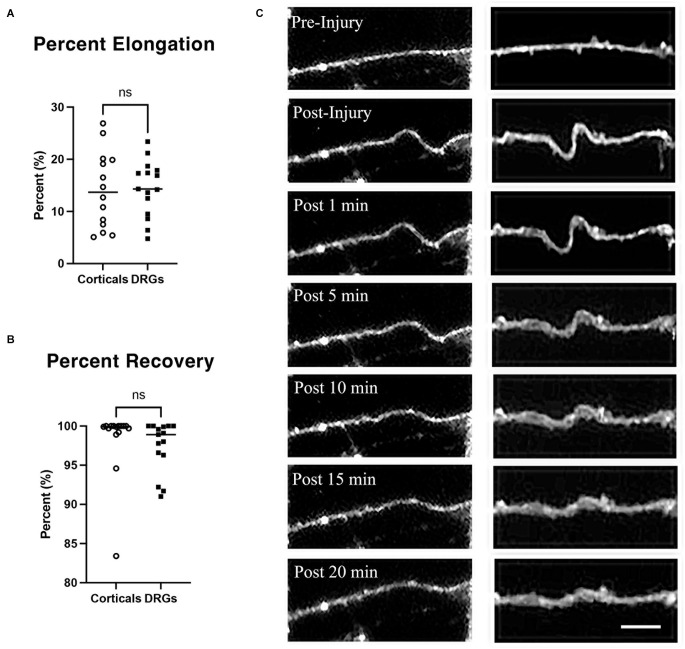
Axon elongation and recovery. **(A)** Cortical and DRGN axons elongated the same amount from a stretch of 60% strain, *p* = 0.90. **(B)** Cortical and DRGN axons recovered similarly 20 min after stretch, *p* = 0.50. Horizontal lines in **(A)** and **(B)** indicate the average of each group. **(C)** Time series of axon undulation and recovery. Axons fluorescently labeled with Fluo-4 AM prior to stretch injury. *Left*: cortical axon, *right*: DRGN axon. Scale bar = 10 μm, ns = not significant.

To quantify the stretch injury-induced lengthening of axons, DRGN, and cortical axons were uniaxially stretch injured with 60% strain at a rate of 30 s^−1^ (*n* = 15 axons). Cultures were labeled with Fluo-4 for fluorescent imaging of axons to enhance contrast. Image J was used to measure axonal lengths prior to injury and immediately after the injury. Cortical axons elongated by 14.14% ± 1.96 over their original lengths. Similarly, DRGNs axons responded to injury with an average elongation of 14.45% ± 1.38 (Figure 2A). The ability of axons to recover to their original length was quantified at 20 min post injury. Cortical axons recovered to 98.35% ± 4.36 of their original length and similarly, DRGNs axons recovered to 97.41 ± 3.23% of their original length (Figures 2B,C). There was no significant difference between the two populations (*p* > 0.05 for elongation and recovery).

### Morphological changes in stretch injured axons

**Figure 3:**
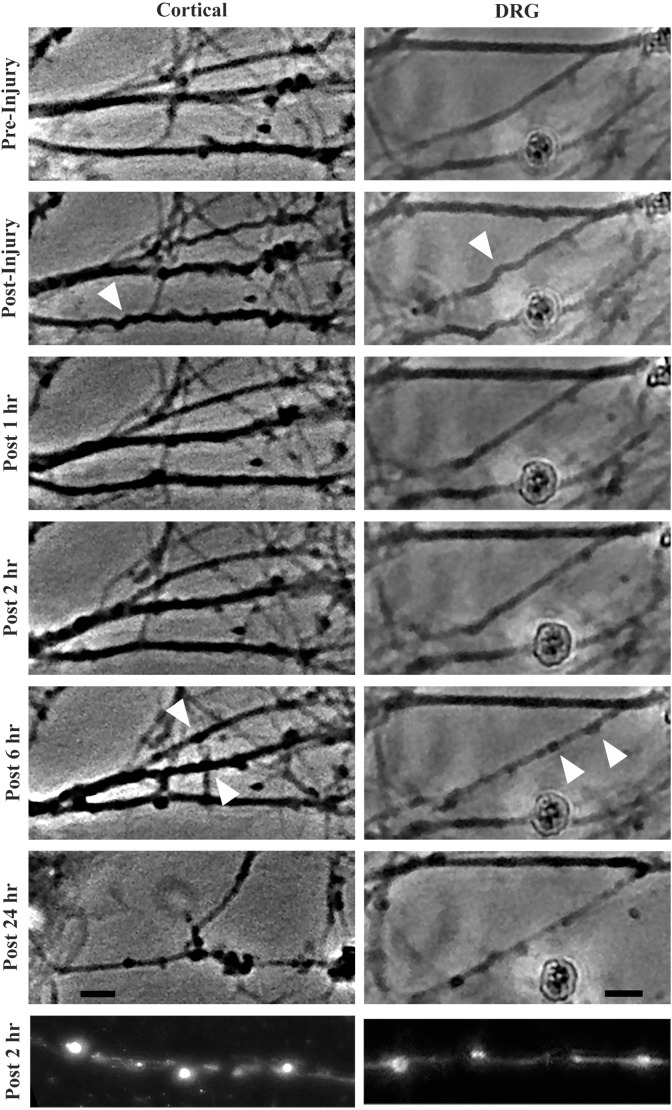
Morphological changes in cortical and DRGN axons after stretch injury of 60% strain. Time course illustrates that undulations are formed after an injury that are fully recovered at 1 h in both cortical and DRGN axons (arrows). At the 2 and 6 h time points, swellings along the axons develop (arrows). After 24 h, axon degeneration and disconnection were identified in both Cortical and DRGN axons. Bottom row, axons labeled for NF-200 show swellings along the axon in both cortical and DRGN axons at 2 h post injury. Scale bars = 10 μm.

Stretch injury severity scales with strain in cultured cortical neurons (Tang-Schomer et al., 2010). To determine how strain magnitude affects morphological outcome in these two neuronal subtypes, cortical and DRGN axons were stretch injured at strains of 40% and 60% at a common strain rate of 30 s^−1^. Axonal swellings have been observed along the length of injured axons in previously described *in vitro* stretch injury models as well as *in vivo* models of TBI. By 2 h post injury, all DRGN and cortical cultures injured at a strain of 60% showed evidence of axonal swellings (Figure 3). Conversely, less than 20% of the DRGN cultures and 50% of the cortical cultures injured at 40% strain developed axonal swellings. Notably, these mildly injured axons that develop undulations did not always develop axonal swellings, suggesting some axons avoid degeneration.

By 4 h post injury, all DRGN and cortical cultures injured at 60% strain displayed axonal degeneration that progressed in severity through the 24-h period of observation. For injuries at 40% strain, only the DRGN cultures that previously developed swellings had observable axonal disconnection by the 4 h post injury time point. 33% of the cortical cultures injured at a strain of 40% were found to have disconnected axons by 24 h post injury, but none of the cultures in the study did at the 4 h time point. This suggests that there may be a delay in this response in cortical axons injured at a lower strain.

### Stretch injury induced calcium influx

**Figure 4:**
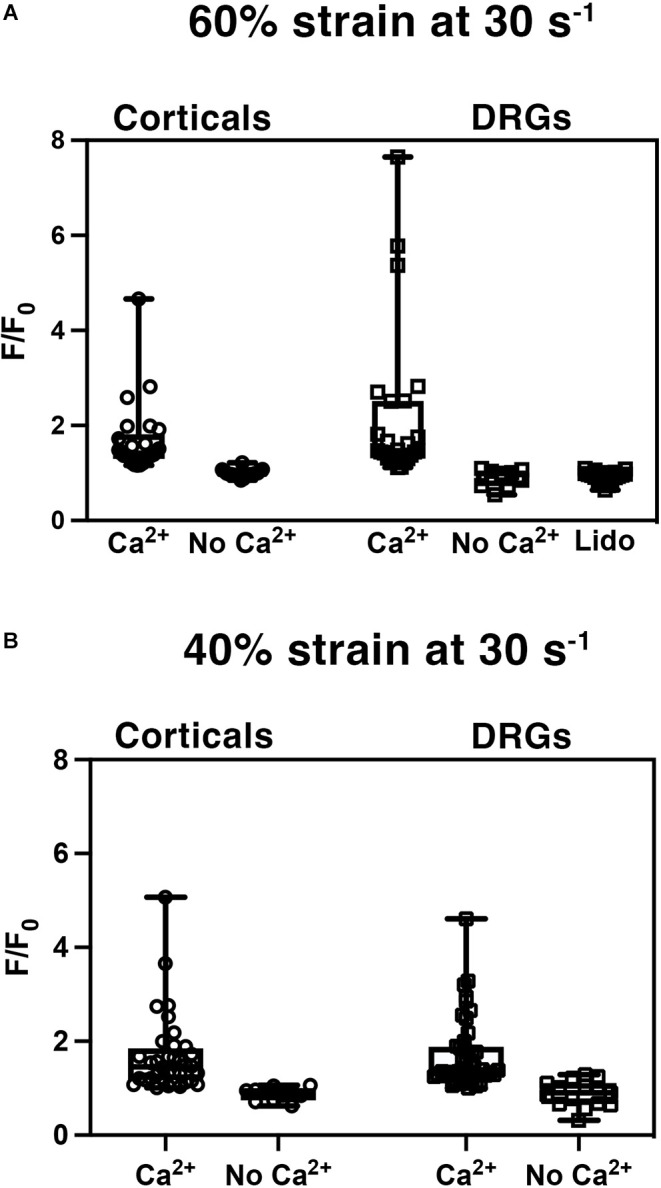
Stretch injury induced increase in intracellular calcium (Ca^2+^). Normalized fluorescent intensity values (F/F_0_) in DRGN and cortical axons after **(A)** injuries at 60% strain at a rate of 30 s^−1^, and **(B)** strain of 40% at a rate of 30 s^−1^. Cultures were injured with the presence of extracellular Ca^2+^ and without -Ca^2+^. DRGN cultures treated with lidocaine (Lido) blocked the injury related influx of calcium.

Cortical and DRGN axons were injured to induce an influx of intracellular calcium, a well-established mechanism in cortical neurons that has been reported to scale with the degree of stretch applied (Lusardi et al., 2004). To test whether this occurs in DRGN, both populations were injured at a strain of 60% at a rate of 30 s^−1^ and a 40% strain at a rate of 30 s^−1^, and the influx of calcium was measured via the change in Fluo-4 fluorescence signal (Figure 4).

Stretch injured cortical axons responded with an increase in Fluo-4 fluorescence to a mean F/F_0_ of 1.68 ± 0.75 (*n* = 25) at 60% strain (Figure 4A). Injured DRGN axons also elicited an increase in Fluo-4 fluorescence to a mean F/F_0_ of 2.20 ± 0.33 (*n* = 25) at 60% strain. To confirm that the rise in intracellular calcium was due to an influx of extracellular calcium, cultures were injured in a calcium-free buffer. Both cortical and DRGN axons injured without the presence of extracellular calcium did not elicit an intracellular rise in calcium, with mean F/F_0_ of 0.99 ± 0.02 (*n* = 19) and 1.03 ± 0.06 (*n* = 9) respectively. *Post hoc* pairwise comparisons indicated that stretch induced calcium influx in DRGN and cortical axons were not significantly different at 60% strain (*p* > 0.05), but were significantly different from controls excluding extracellular calcium (*p* < 0.05).

Blocking sodium channels with tetrodotoxin (TTX) in cortical axons prevents calcium entry after injury (Wolf et al., 2001). Unlike cortical neurons, DRGN have TTX resistant sodium channels. To test the mechanism of sodium channel blockage on calcium influx in DRGNs axons, we stretch injured axons at 60% strain, 30 s^−1^ strain rate in the presence of lidocaine (Roy and Narahashi, 1992). DRGN cultures that received lidocaine treatment prior to injury (*n* = 18) did not respond with an influx of calcium (F/F_0_ = 0.93 ± 0.03; Figure 4A). Lidocaine treatment leads to calcium measurements similar to controls (*p* = 1 with Bonferroni adjustment) and significantly different from untreated DRGN axons (*p* < 0.05).

To investigate whether calcium influx into axons scaled with the level of injury, cortical and DRGN axons injured at 60% strain were compared to axons injured at 40% strain. Stretch injured cortical axons responded with an increase in Fluo-4 fluorescence to a mean F/F_0_ of 1.68 ± 0.14 (*n* = 36) at 40% strain, 0.88 ± 0.04 (*n* = 11) in buffer without calcium (Figure 4B). Injury to DRGN axons also elicited an increase in Fluo-4 fluorescence to a mean F/F_0_ of 1.72 ± 0.11 (*n* = 44) at 40% strain, 0.89 ± 0.07 (*n* = 15) in buffer without calcium. DRGN and cortical groups injured at 60% and 40% strain were not significantly different (*p* > 0.05). This data also indicates that calcium influx levels in both DRGN and cortical axons do not scale with the degree of stretch.

### Sodium channel proteolysis in stretch-injured DRGN axons

**Figure 5:**
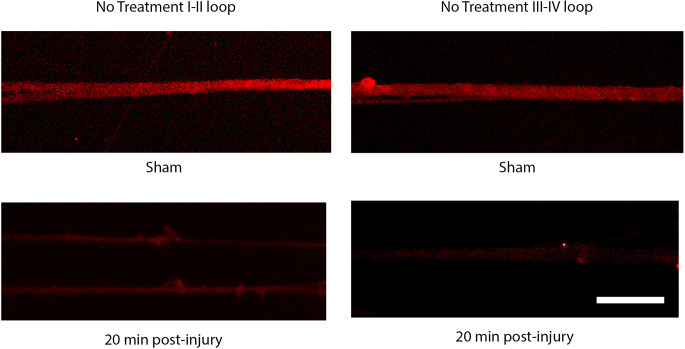
Immunoreactivity of the I-II loop and III-IV loops of the sodium channel. The III-IV loop is cleaved by calcium activated proteases and is lost at 20 post injury. The I-II loop is retained within the cell membrane and is present at 20 post injury. Scale bar = 10 μm.

**Figure 6:**
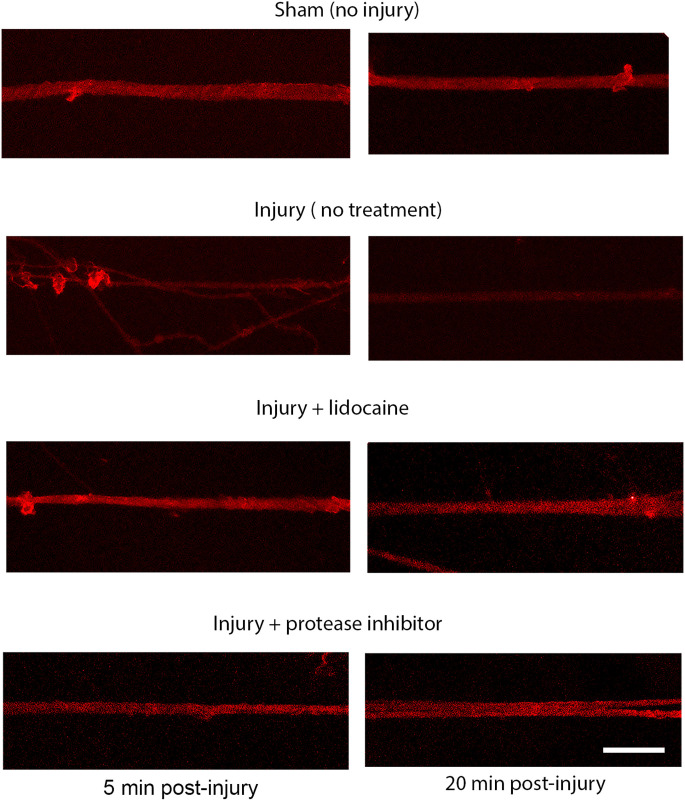
Proteolysis of the sodium channel III-IV loop post injury in DRGN axons. Expression of the NaCh III-IV loop after 60% stretch injury is attenuated at 5 min and lost completely by 20 min. Blocking sodium channels with lidocaine prevents the cleavage of the III-IV loop. Cleavage is also prevented by blocking protease activity. This mechanism is similar to the response in injured cortical neurons. Scale bar = 10 μm.

The injury induced calcium influx activates proteases that cleave the III-IV intracellular loop of the sodium channel while the I-II loop remains in the membrane of cortical axons (Iwata et al., 2004). Injured DRGN axons were found to respond similarly where the NaCh I-II loop was retained in injured axons and expression of the III-IV is undetectable within the first 20 min after injury (Figure 5). Pre-treatment with either lidocaine or broad-spectrum protease inhibitors prevented the cleavage of the intracellular III-IV loop and retained after injury in DRGN axons (Figure 6).

## Discussion

Traumatic brain injury (TBI) is initiated by a traumatic mechanical loading to the head that translates to rapid mechanical stretching of neurons and their axons. The injurious mechanical stretch initiates biochemical sequalae that propagates secondary injury mechanisms. It is difficult to elucidate how the dynamic deformation of neuronal cells evolves into dysfunction and degeneration using *in vivo* models. Many groups utilize an *in vitro* stretch injury model to directly study biomechanical mechanisms of injury to neurons *in real time.* These models have used cortical and hippocampal neuronal cultures that can replicate many of the morphological and ultrastructural changes observed *in vivo* including alterations in axolemmal permeability, pathology associated with diffuse axonal injury (DAI), and neural degeneration (Smith et al., 1999; Smith and Meaney, 2000; Pfister et al., 2003, 2004; Magou et al., 2011, 2015).

Interestingly, the progression of neuronal degeneration is quite similar among neurological diseases, injury, and neuronal cell type. Known molecular mechanisms of axonal degeneration from mechanical injury in TBI are common to Alzheimer’s disease, ischemia and spinal cord injury (SCI). For instance, proteolysis of sodium channels is shared with ischemic models (Stys et al., 1992; Stys, 1998). Changes in beta amyloid precursor protein present a potential link between TBI and Alzheimer’s disease (Friedlander, 2003; Chen et al., 2004; Johnson et al., 2010; Wang et al., 2012). Mechanical stretch to DRGN axons has been used to model the effects of long-term stretch in relationship to SCI (Blight and Decrescito, 1986; Blight, 1988; Shi and Pryor, 2002; Friedlander, 2003; Gladman et al., 2010; Ye et al., 2012). The ensuing injury response has similarities to TBI including sustained rise in intracellular calcium, activation of caspases and calpain, cytoskeletal breakdown, and axonal disconnection. Accordingly, we hypothesized that DRGN axons would exhibit a similar response to stretch injury compared to cortical neurons.

Cortical and hippocampal cultures have limited ability to address some experimental questions in relation to TBI. The mixed embryonic cortical cultures used for stretch injury do not exhibit longevity in culture. In our experience cultures are not reliable past 14 DIV and studies using a stretch injury model are performed prior to 14 DIV (Wu et al., 2021). Cortical cultures are not myelinated whereas DAI is a pathology that occurs primarily in white matter tracts. Unfortunately, cortical cultures are exceedingly difficult to myelinate in culture and therefore unrealistic for use in a stretch injury model (Kim et al., 2007; Schonfeld-Dado et al., 2009). DRGNs are a neuronal cell type that can be maintained in culture over longer periods of time and are capable of being myelinated by CNS myelinating glia (Stevens et al., 2002; Wake et al., 2011; Lundgaard et al., 2013). The goal of this investigation was to identify whether DRGNs can serve as a viable model to study the cellular events initiated by mechanical stretch and the associated secondary injury mechanisms.

DRGNs, however, have distinct differences from brain derived neurons including voltage gated ion channel expression, signaling receptors, and electrophysiological activity (Rizzo et al., 1994; Scholz and Vogel, 2000). There are also large known differences in the regenerative response of PNS and CNS axons to injury including fundamental differences in cytoskeletal and transport dynamics (Liu and Brady, 2004; Loverde et al., 2020). It is currently unknown if DRGNs would respond similarly to a traumatic stretch injury as cortical cultures and allow for the study of TBI mechanisms. Here we show that cortical and DRGN axons experience similar morphological alterations within the first 20 min after severe (60%) stretch injury, as well as comparable patterns of degeneration over the course of 24 h. Under 40% strain, however, we found differences in their response. At 40% strain, fewer DRGN axons developed axonal swellings compared to cortical axons. Of these axons developing swellings, DRGN axon disconnection from degeneration was observed as early as 4 h whereas cortical axons disconnection occurred after 4 h. Importantly, moderately injured axons that develop undulations may not always commit to degeneration and develop axonal swellings, suggesting some axons recover. This has been speculated in other work (Smith and Meaney, 2000).

Live calcium imaging revealed a comparable degree of intracellular influx of calcium in both neurons and did not differ in regards to the degree of stretch. The results also reveal loss of the sodium channel III-IV intracellular loop in stretch injured DRGN axons that is attenuated by pre-treatment with the broad-spectrum sodium channel antagonist, lidocaine. Accordingly, elevated calcium in axons from stretch may not directly correspond to degeneration.

While both neuronal sources seem to respond similarly to mechanical stretch, there are differences in the neuron types and culture conditions that need to be considered. DRGN required a basement membrane of Matrigel to adhere to the silicone substrate whereas for cortical neurons PDL was sufficient. We do not believe these differences would affect the mechanical initiation of injury as indicated by the influx of calcium and morphological response in terms of undulation formation and retraction. The development of axonal swellings and disconnection between the neuronal types at 24 h after moderate (40% stretch) injury, however, suggests potential differences in the neurodegenerative process. DRGNs have been shown to tolerate slow mechanical stretch better than cortical axons in a different model of stretch induced growth (Loverde et al., 2020). DRGNs also have the addition of NGF which has been considered neuroprotective (Tanaka et al., 1990; Vogelbaum et al., 1998; Liu and Li, 2009). Less obvious structural changes to DRGN axons may also be related to the population of axons in culture, as it is more difficult to isolate individual axons due to the size and robust nature of peripheral axons (Debanne et al., 2011).

Using cortical neurons, it has been shown that rapid mechanical stretch leads to an influx of extracellular calcium that is mediated by the sodium channel; a process that can be blocked with TTX. It is hypothesized that mechanical stretch causes a loss in sodium homeostasis that leads to an overload of calcium as the neuron attempts to restore the sodium gradient. The elevated calcium activates calcium mediated proteases leading to the cleavage of the sodium channel III-IV loop considered to be the channel inactivation gate, perpetuating the imbalance in ion homeostasis (Wolf et al., 2001; Iwata et al., 2004). While both neurons responded similarly to the stretch induced influx of calcium, DRGN expresses different subtypes of sodium channels including TTX insensitive channels whereas cortical neurons in culture express primarily TTX sensitive subtypes (Catterall et al., 2005; Ho and O’Leary, 2011; de Lera Ruiz and Kraus, 2015). In this study, the blockage of sodium channels in DRGN axons with lidocaine prevented injury induced calcium influx. Lidocaine, however, is known to be a broad channel blocker, suggesting that the injury mechanism may be similar to other channel subtypes (Kostyuk, 1981; Roy and Narahashi, 1992). Previously, it has not been demonstrated whether blocking sodium channels in the DRGN axon is sufficient to prevent calcium influx and sodium channel proteolysis post stretch injury. Future work may consider if injury induced proteolysis affects other sodium channel subtypes and the implications to neuronal function.

An important finding from this study is the similarity between the calcium influx at both 60 and 40% strains. This suggests that in axons, the degree of calcium influx does not scale with the magnitude of the injury. Indeed, there may be an injury threshold at which axons accumulate intracellular calcium (Yuen et al., 2009). Previous work showing intracellular calcium accumulation scales with injury were measured in the soma compartment, a process that has been shown to include calcium overload due to synaptic receptors in addition to sodium channels (Zhang et al., 1996; Lea et al., 2003; Spaethling et al., 2008). Further studies are needed to determine the link between the mechanical changes to the sodium channel, changes in intracellular sodium, and the ensuing rapid change in intracellular calcium.

This study compares the response of DRGN axons to cortical axons commonly used in an *in vitro* stretch injury model of TBI. The results demonstrate that important initial injury mechanisms related to a rapid mechanical stretch are shared between DRGN and cortical axons. While DRGNs may offer the ability to study *in vitro* injury experiments where cortical cultures fall short, DRGNs can be also isolated and cultured from adult animals and humans (Huang et al., 2008; Loverde et al., 2011). There are well-known differences in the response of embryonic and adult neurons to injury and has been a major criticism of the clinical relevance of* in vitro* TBI models (Wu et al., 2021). DRGN may allow future studies to explore TBI injury progression in adult neurons.

## Data availability statement

The raw data supporting the conclusions of this article will be made available by the authors, without undue reservation.

## Ethics statement

The animal study was reviewed and approved by The Rutgers University IACUC.

## Author contributions

BP, AA, and HK conceived and planned the experiments. AA and YL performed the experiments. BP, AA, YL, and HK contributed to the interpretation of the results and presentation of the data. BP and AA wrote the manuscript. BP and HK were in charge of overall direction, planning and funding of the work. All authors contributed to the article and approved the submitted version.

## Funding

This work was supported by the New Jersey Commission on Brain Injury Research CBIR16PIL018 to BP and NIH R21NS109708 to HK.

## Conflict of Interest

The remaining authors declare that the research was conducted in the absence of any commercial or financial relationships that could be construed as a potential conflict of interest.

## Publisher’s note

All claims expressed in this article are solely those of the authors and do not necessarily represent those of their affiliated organizations, or those of the publisher, the editors and the reviewers. Any product that may be evaluated in this article, or claim that may be made by its manufacturer, is not guaranteed or endorsed by the publisher.

## Abbreviations

DRGN: dorsal root ganglia neurons
TBI: traumatic brain injury
DAI: diffuse axonal injury
DIV: days *in vitro*
TTX: tetrodotoxin.

## References

[B1] Adams J. H. Doyle D. Graham D. I. Lawrence A. E. McLellan D. R. (1984). Diffuse axonal injury in head injuries caused by a fall. Lancet 2, 1420–1422. 10.1016/s0140-6736(84)91620-9 6151042

[B2] Blight A. (1988). Mechanical factors in experimental spinal cord injury. J. Am. Paraplegia Soc. 11, 26–34. 10.1080/01952307.1988.11735792 3076595

[B3] Blight A. R. Decrescito V. (1986). Morphometric analysis of experimental spinal cord injury in the cat: the relation of injury intensity to survival of myelinated axons. Neuroscience 19, 321–341. 10.1016/0306-4522(86)90025-4 3785669

[B4] Cargill R. S. 2nd Thibault L. E. (1996). Acute alterations in [Ca^2+^]i in NG108-15 cells subjected to high strain rate deformation and chemical hypoxia: an *in vitro* model for neural trauma. J. Neurotrauma 13, 395–407. 10.1089/neu.1996.13.395 8863195

[B5] Catterall W. A. Goldin A. L. Waxman S. G. (2005). International union of pharmacology. XLVII. Nomenclature and structure-function relationships of voltage-gated sodium channels. Pharmacol. Rev. 57, 397–409. 10.1124/pr.57.4.4 16382098

[B6] Chen X.-H. Siman R. Iwata A. Meaney D. F. Trojanowski J. Q. Smith D. H. (2004). Long-term accumulation of amyloid-β, β-secretase, presenilin-1 and caspase-3 in damaged axons following brain trauma. Am. J. Pathol. 165, 357–371. 10.1016/s0002-9440(10)63303-2 15277212 PMC1618579

[B7] Cullen D. K. Vernekar V. N. LaPlaca M. C. (2011). Trauma-induced plasmalemma disruptions in three-dimensional neural cultures are dependent on strain modality and rate. J. Neurotrauma 28, 2219–2233. 10.1089/neu.2011.1841 22023556 PMC3218387

[B8] de Lera Ruiz M. Kraus R. L. (2015). Voltage-gated sodium channels: structure, function, pharmacology and clinical indications. J. Med. Chem. 58, 7093–7118. 10.1021/jm501981g 25927480

[B9] Debanne D. Campanac E. Bialowas A. Carlier E. Alcaraz G. (2011). Axon physiology. Physiol. Rev. 91, 555–602. 10.1152/physrev.00048.2009 21527732

[B10] Di X. Goforth P. B. Bullock R. Ellis E. Satin L. (2000). Mechanical injury alters volume activated ion channels in cortical astrocytes. Acta Neurochir. Suppl. 76, 379–383. 10.1007/978-3-7091-6346-7_79 11450049

[B11] Ellis E. F. McKinney J. S. Willoughby K. A. Liang S. Povlishock J. T. (1995). A new model for rapid stretch-induced injury of cells in culture: characterization of the model using astrocytes. J. Neurotrauma 12, 325–339. 10.1089/neu.1995.12.325 7473807

[B12] Friedlander R. M. (2003). Apoptosis and caspases in neurodegenerative diseases. N. Engl. J. Med. 348, 1365–1375. 10.1056/NEJMra022366 12672865

[B13] Galbraith J. A. Thibault L. E. Matteson D. R. (1993). Mechanical and electrical responses of the squid giant axon to simple elongation. J. Biomech. Eng. 115, 13–22. 10.1115/1.2895464 8445893

[B14] Gladman S. J. Ward R. E. Michael-Titus A. T. Knight M. M. Priestley J. V. (2010). The effect of mechanical strain or hypoxia on cell death in subpopulations of rat dorsal root ganglion neurons in vitro. Neuroscience 171, 577–587. 10.1016/j.neuroscience.2010.07.009 20674687

[B15] Goforth P. B. Ellis E. F. Satin L. S. (2004). Mechanical injury modulates AMPA receptor kinetics via an NMDA receptor-dependent pathway. J. Neurotrauma 21, 719–732. 10.1089/0897715041269704 15253800

[B16] Grady M. S. McLaughlin M. R. Christman C. W. Valadka A. B. Fligner C. L. Povlishock J. T. (1993). The use of antibodies targeted against the neurofilament subunits for the detection of diffuse axonal injury in humans. J. Neuropathol. Exp. Neurol. 52, 143–152. 10.1097/00005072-199303000-00007 8440996

[B17] Heffernan C. Maurel P. (2018). Lentiviral transduction of rat schwann cells and dorsal root ganglia neurons for *in vitro* myelination studies. Methods Mol. Biol. 1739, 177–193. 10.1007/978-1-4939-7649-2_12 29546708

[B18] Ho C. O’Leary M. E. (2011). Single-cell analysis of sodium channel expression in dorsal root ganglion neurons. Mol. Cell. Neurosci. 46, 159–166. 10.1016/j.mcn.2010.08.017 20816971 PMC3005531

[B19] Hoffman P. N. (2010). A conditioning lesion induces changes in gene expression and axonal transport that enhance regeneration by increasing the intrinsic growth state of axons. Exp. Neurol. 223, 11–18. 10.1016/j.expneurol.2009.09.006 19766119

[B20] Holbourn A. H. S. (1945). The mechanics of brain injuries. Br. Med. Bull. 3, 147–149. 10.1093/oxfordjournals.bmb.a071895

[B21] Huang J. H. Cullen D. K. Browne K. D. Groff R. Zhang J. Pfister B. J.. (2009). Long-term survival and integration of transplanted engineered nervous tissue constructs promotes peripheral nerve regeneration. Tissue Eng. Part A 15, 1677–1685. 10.1089/ten.tea.2008.0294 19231968 PMC2792099

[B22] Huang J. H. Zager E. L. Zhang J. Groff R. F. Pfister B. J. Cohen A. S.. (2008). Harvested human neurons engineered as live nervous tissue constructs: implications for transplantation. Laboratory investigation. J. Neurosurg. 108, 343–347. 10.3171/JNS/2008/108/2/0343 18240932 PMC3014262

[B23] Iwata A. Stys P. K. Wolf J. A. Chen X.-H. Taylor A. G. Meaney D. F.. (2004). Traumatic axonal injury induces proteolytic cleavage of the voltage-gated sodium channels modulated by tetrodotoxin and protease inhibitors. J. Neurosci. 24, 4605–4613. 10.1523/JNEUROSCI.0515-03.2004 15140932 PMC6729402

[B24] Johnson V. E. Stewart W. Smith D. H. (2010). Traumatic brain injury and amyloid-β pathology: a link to Alzheimer’s disease? Nat. Rev. Neurosci. 11, 361–370. 10.1038/nrn2808 20216546 PMC3979339

[B25] Johnson V. E. Stewart W. Smith D. H. (2013). Axonal pathology in traumatic brain injury. Exp. Neurol. 246, 35–43. 10.1016/j.expneurol.2012.01.013 22285252 PMC3979341

[B26] Kao C. Q. Goforth P. B. Ellis E. F. Satin L. S. (2004). Potentiation of GABA(A) currents after mechanical injury of cortical neurons. J. Neurotrauma 21, 259–270. 10.1089/089771504322972059 15115601

[B27] Kim M. J. Oh S. J. Park S. H. Kang H. J. Won M. H. Kang T. C.. (2007). Neuronal loss in primary long-term cortical culture involves neurodegeneration-like cell death via calpain and p35 processing, but not developmental apoptosis or aging. Exp. Mol. Med. 39, 14–26. 10.1038/emm.2007.3 17334225

[B28] Kostyuk G. P. (1981). Calcium channels in the neuronal membrane. Biochim. Biophys. Acta 650, 128–150. 10.1016/0304-4157(81)90003-4 6277370

[B29] LaPlaca M. C. Thibault L. E. (1997). An in vitro traumatic injury model to examine the response of neurons to a hydrodynamically-induced deformation. Ann. Biomed. Eng. 25, 665–677. 10.1007/BF02684844 9236979

[B30] Lea P. M. IV Custer S. J. Stoica B. A. Faden A. I. (2003). Modulation of stretch-induced enhancement of neuronal NMDA receptor current by mGluR1 depends upon presence of glia. J. Neurotrauma 20, 1233–1249. 10.1089/089771503770802907 14651810

[B31] Liu H. H. Brady S. T. (2004). cAMP, tubulin, axonal transport and regeneration. Exp. Neurol. 189, 199–203. 10.1016/j.expneurol.2004.06.006 15380472

[B32] Liu R. Lin G. Xu H. (2013). An efficient method for dorsal root ganglia neurons purification with a one-time anti-mitotic reagent treatment. PLoS One 8:e60558. 10.1371/journal.pone.0060558 23565257 PMC3614500

[B33] Liu Z. Li Z. (2009). Nerve growth factor and norepinephrine regulate galanin expression in primary cultures of dorsal root ganglion neurons. Pharm. Biol. 47, 634–639. 10.1080/13880200902915630

[B34] Loverde J. R. Ozoka V. C. Aquino R. Lin L. Pfister B. J. (2011). Live imaging of axon stretch growth in embryonic and adult neurons. J. Neurotrauma 28, 2389–2403. 10.1089/neu.2010.1598 21663384

[B35] Loverde J. R. Tolentino R. E. Soteropoulos P. Pfister B. J. (2020). Biomechanical forces regulate gene transcription during stretch-mediated growth of mammalian neurons. Front. Neurosci. 14:600136. 10.3389/fnins.2020.600136 33408609 PMC7780124

[B37] Lundgaard I. Luzhynskaya A. Stockley J. H. Wang Z. Evans K. A. Swire M.. (2013). Neuregulin and BDNF induce a switch to NMDA receptor-dependent myelination by oligodendrocytes. PLoS Biol. 11:e1001743. 10.1371/journal.pbio.1001743 24391468 PMC3876980

[B38] Lusardi T. A. Wolf J. A. Putt M. E. Smith D. H. Meaney D. F. (2004). Effect of acute calcium influx after mechanical stretch injury in vitro on the viability of hippocampal neurons. J. Neurotrauma 21, 61–72. 10.1089/089771504772695959 14987466

[B39] Magou G. C. Guo Y. Choudhury M. Chen L. Hususan N. Masotti S.. (2011). Engineering a high throughput axon injury system. J. Neurotrauma 28, 2203–2218. 10.1089/neu.2010.1596 21787172

[B40] Magou G. C. Pfister B. J. Berlin J. R. (2015). Effect of acute stretch injury on action potential and network activity of rat neocortical neurons in culture. Brain Res. 1624, 525–535. 10.1016/j.brainres.2015.07.056 26296661

[B41] Monnerie H. Tang-Schomer M. D. Iwata A. Smith D. H. Kim H. A. Le Roux P. D. (2010). Dendritic alterations after dynamic axonal stretch injury *in vitro*. Exp. Neurol. 224, 415–423. 10.1016/j.expneurol.2010.05.001 20478308 PMC3979358

[B42] Morrison B. 3rd Cater H. L. Benham C. D. Sundstrom L. E. (2006). An *in vitro* model of traumatic brain injury utilising two-dimensional stretch of organotypic hippocampal slice cultures. J. Neurosci. Methods 150, 192–201. 10.1016/j.jneumeth.2005.06.014 16098599

[B43] Morrison B. 3rd Elkin B. S. Dolle J. P. Yarmush M. L. (2011). *in vitro* models of traumatic brain injury. Annu. Rev. Biomed. Eng. 13, 91–126. 10.1146/annurev-bioeng-071910-124706 21529164

[B45] Pfister B. Oyler G. Betenbaugh M. Bao G. (2004). The effects of BclXL and Bax over-expression on stretch-injury induced neural cell death. Mech. Chem. Biosyst. 1, 233–243. 10.3970/mcb.2004.001.233 16783920

[B44] Pfister B. J. Weihs T. P. Betenbaugh M. Bao G. (2003). An *in vitro* uniaxial stretch model for axonal injury. Ann. Biomed. Eng. 31, 589–598. 10.1114/1.1566445 12757202

[B46] Rizzo M. A. Kocsis J. D. Waxman S. G. (1994). Slow sodium conductances of dorsal root ganglion neurons: intraneuronal homogeneity and interneuronal heterogeneity. J. Neurophysiol. 72, 2796–2815. 10.1152/jn.1994.72.6.2796 7897490 PMC2605955

[B47] Roy M. L. Narahashi T. (1992). Differential properties of tetrodotoxin-sensitive and tetrodotoxin-resistant sodium channels in rat dorsal root ganglion neurons. J. Neurosci. 12, 2104–2111. 10.1523/JNEUROSCI.12-06-02104.1992 1318956 PMC6575921

[B48] Schneider C. A. Rasband W. S. Eliceiri K. W. (2012). NIH image to imageJ: 25 years of image analysis. Nat. Methods 9, 671–675. 10.1038/nmeth.2089 22930834 PMC5554542

[B49] Scholz A. Vogel W. (2000). Tetrodotoxin-resistant action potentials in dorsal root ganglion neurons are blocked by local anesthetics. Pain 89, 47–52. 10.1016/S0304-3959(00)00345-6 11113292

[B50] Schonfeld-Dado E. Fishbein I. Segal M. (2009). Degeneration of cultured cortical neurons following prolonged inactivation: molecular mechanisms. J. Neurochem. 110, 1203–1213. 10.1111/j.1471-4159.2009.06204.x 19508430

[B51] Shi R. Pryor J. D. (2002). Pathological changes of isolated spinal cord axons in response to mechanical stretch. Neuroscience 110, 765–777. 10.1016/s0306-4522(01)00596-6 11934483

[B52] Smith D. H. Meaney D. F. (2000). Axonal damage in traumatic brain injury. Neuroscientist 6, 483–495. 10.1177/107385840000600611

[B53] Smith D. H. Meaney D. F. Shull W. H. (2003). Diffuse axonal injury in head trauma. J. Head Trauma Rehabil. 18, 307–316. 10.1097/00001199-200307000-00003 16222127

[B54] Smith D. H. Wolf J. A. Lusardi T. A. Lee V. M. Meaney D. F. (1999). High tolerance and delayed elastic response of cultured axons to dynamic stretch injury. J. Neurosci. 19, 4263–4269. 10.1523/JNEUROSCI.19-11-04263.1999 10341230 PMC6782601

[B55] Spaethling J. M. Klein D. M. Singh P. Meaney D. F. (2008). Calcium-permeable AMPA receptors appear in cortical neurons after traumatic mechanical injury and contribute to neuronal fate. J. Neurotrauma 25, 1207–1216. 10.1089/neu.2008.0532 18986222 PMC2799682

[B56] Stevens B. Porta S. Haak L. L. Gallo V. Fields R. D. (2002). Adenosine: a neuron-glial transmitter promoting myelination in the CNS in response to action potentials. Neuron 36, 855–868. 10.1016/s0896-6273(02)01067-x 12467589 PMC1201407

[B57] Stys P. K. (1998). Anoxic and ischemic injury of myelinated axons in CNS white matter: from mechanistic concepts to therapeutics. J. Cereb. Blood Flow Metab. 18, 2–25. 10.1097/00004647-199801000-00002 9428302

[B58] Stys P. K. Waxman S. G. Ransom B. R. (1992). Ionic mechanisms of anoxic injury in mammalian CNS white matter: role of Na^+^ channels and Na^+^-Ca^2+^ exchanger. J. Neurosci. 12, 430–439. 10.1523/JNEUROSCI.12-02-00430.1992 1311030 PMC6575619

[B59] Tanaka M. Kanai H. Hirai S. (1990). Effects of nerve growth factor on cultured adult dorsal root ganglion neurons evaluated by enzyme immunoassay for neurofilament protein. AGE 13, 57–60. 10.1007/BF02432390

[B60] Tang-Schomer M. D. Patel A. R. Baas P. W. Smith D. H. (2010). Mechanical breaking of microtubules in axons during dynamic stretch injury underlies delayed elasticity, microtubule disassembly and axon degeneration. FASEB J. 24, 1401–1410. 10.1096/fj.09-142844 20019243 PMC2879950

[B61] Tavalin S. J. Ellis E. F. Satin L. S. (1997). Inhibition of the electrogenic Na pump underlies delayed depolarization of cortical neurons after mechanical injury or glutamate. J. Neurophysiol. 77, 632–638. 10.1152/jn.1997.77.2.632 9065836

[B62] Thévenaz P. Ruttimann U. E. Unser M. (1998). A pyramid approach to subpixel registration based on intensity. IEEE Trans. Image Process. 7, 27–41. 10.1109/83.650848 18267377

[B63] Vogelbaum M. A. Tong J. X. Rich K. M. (1998). Developmental regulation of apoptosis in dorsal root ganglion neurons. J. Neurosci. 18, 8928–8935. 10.1523/JNEUROSCI.18-21-08928.1998 9786998 PMC6793517

[B64] Wake H. Lee P. R. Fields R. D. (2011). Control of local protein synthesis and initial events in myelination by action potentials. Science 333, 1647–1651. 10.1126/science.1206998 21817014 PMC3482340

[B65] Wang J. T. Medress Z. A. Barres B. A. (2012). Axon degeneration: molecular mechanisms of a self-destruction pathway. J. Cell Biol. 196, 7–18. 10.1083/jcb.201108111 22232700 PMC3255986

[B66] Wolf J. A. Stys P. K. Lusardi T. Meaney D. Smith D. H. (2001). Traumatic axonal injury induces calcium influx modulated by tetrodotoxin-sensitive sodium channels. J. Neurosci. 21, 1923–1930. 10.1523/JNEUROSCI.21-06-01923.2001 11245677 PMC6762603

[B67] Wu Y. H. Rosset S. Lee T. R. Dragunow M. Park T. Shim V. (2021). in vitro models of traumatic brain injury: a systematic review. J. Neurotrauma 38, 2336–2372. 10.1089/neu.2020.7402 33563092

[B68] Ye Z. Wang Y. Quan X. Li J. Hu X. Huang J.. (2012). Effects of mechanical force on cytoskeleton structure and calpain-induced apoptosis in rat dorsal root ganglion neurons *in vitro*. PLoS One 7:e52183. 10.1371/journal.pone.0052183 23284927 PMC3527405

[B69] Yuen T. J. Browne K. D. Iwata A. Smith D. H. (2009). Sodium channelopathy induced by mild axonal trauma worsens outcome after a repeat injury. J. Neurosci. Res. 87, 3620–3625. 10.1002/jnr.22161 19565655 PMC3014254

[B70] Zhang L. Rzigalinski B. A. Ellis E. F. Satin L. S. (1996). Reduction of voltage-dependent Mg^2+^ blockade of NMDA current in mechanically injured neurons. Science 274, 1921–1923. 10.1126/science.274.5294.1921 8943207

